# Engineered biochar from wood apple shell waste for high-efficient removal of toxic phenolic compounds in wastewater

**DOI:** 10.1038/s41598-021-82277-2

**Published:** 2021-01-28

**Authors:** Nadavala Siva Kumar, Hamid M. Shaikh, Mohammad Asif, Ebrahim H. Al-Ghurabi

**Affiliations:** 1 Department of Chemical Engineering, King Saud University, P.O. Box 800, Riyadh, 11421 Saudi Arabia; 2 Department of Chemical Engineering, SABIC Polymer Research Centre, King Saud University, P.O. Box 800, Riyadh, 11421 Saudi Arabia

**Keywords:** Environmental sciences, Analytical chemistry, Chemical engineering, Environmental chemistry, Surface chemistry

## Abstract

This study investigated a novel agricultural low-cost bio-waste biochar derived from wood apple fruit shell waste via the pyrolysis method, which is modified by ball milling and utilized to remove toxic phenol and chlorophenols (4-CPh and 2,4-DCPh) from contaminated aqueous media. The ball-milled wood apple fruit shell waste biochar (WAS-BC) sorbent was systematically analyzed by BET, CHN, and FTIR as well as particle size, SEM–EDS, XPS and TGA studies. The sorption equilibrium and kinetic studies exhibit that the sorption capacity was greater than 75% within the first 45 min of agitation at pH 6.0. The uptake capacity of 2,4-DCPh onto WAS-BC was greater than those of 4-CPh and phenol. Equilibrium results were consistent with the Langmuir isotherm model, while the kinetic data were best represented by the Elovich and pseudo-second-order model. The maximum uptake of phenol, 4-CPh, and 2,4-DCPh was 102.71, 172.24, and 226.55 mg/g, respectively, at 30 ± 1 °C. Thus, this study demonstrates that WAS-BC is an efficient, low-cost sorbent that can be used for the elimination of phenol and chlorophenol compounds from polluted wastewater.

## Introduction

Water is an essential natural resource; it is required for the entire human, fauna, and flora life cycle. Environmental water contamination is becoming the most important problem of the twenty-first century owing to the rapid development of several industrial activities^1–4^. The progressive development of industries has produced many new products and facilities but also resulted in contamination by toxic inorganic and organic compounds. Chemical and petrochemical industries have been recognized as the main sources for producing organic contaminants. Phenolic organic compounds (e.g., phenol, 4-chlorophenol, and 2,4-dichlorophenol) are the typical constituents of industrial wastewater generated from phenolic resin, plastics manufacturing, petroleum refinery, dye, textile, agrochemical, wood, pesticide, paper, and pharmaceutical industries^5,6^. The existing organic pollutants in various groundwater or surface waters considerably affect the toxicity of water resources and without treatment may present serious health hazards to animals, humans, and aquatic environments^7,8^. Ingestion of water containing phenol and its derivative compounds may cause liver and pancreas damage, kidney failure, and paralysis of the nervous system^9–11^. Owing to the high toxicity and persistent properties of contaminants, these compounds need to be eliminated before being released into water bodies.

The development of new efficient methods for phenolic remediation from polluted water is still an important topic. Thus far, several treatment methods including biological degradation^12^, photocatalytic degradation^13^, electrochemical oxidation^14^, membrane filtration^15^, solvent extraction^16^, and adsorption/biosorption^17–19^ methods have been recommended for the elimination of phenolic compounds from polluted waters. Among these techniques, adsorption technology is broadly used compared to other approaches owing to its easy operation, economical advantages, and greater efficiency of removing inorganic and organic contaminants on industrial or laboratory scales.

Different agro-waste or other biomass waste added-value materials for the removal of phenol and its derivative compounds contained in aqueous wastewaters have not been thoroughly examined. Therefore, recently, many researchers have focused their efforts on enhancing the adsorption capacity using different carbon-rich adsorbent materials such as activated carbon^20–22^, activated carbon fibers^23^, carbon nanocomposites^24,25^, activated biochar-supported magnetite composite and activated biochar^26,27^, carbon nanotubes^28,29^, graphene oxide^30,31^, and biochar^9,32^ to eliminate phenolic contaminants from wastewater. Activated carbon is one of the most widely used sorbents for the adsorption technique^33^. Nevertheless, it has certain disadvantages, e.g., high cost, multistep preparation process, comparatively slow pollutant removal, and often poor regeneration performance^34^.

Recent studies have shown that various forms of bio based carbon materials are highly effective for utilizing in various applications^35–39^. Specifically, attention has been paid to various aspects of biochar preparation to use their favorable characteristics for the adsorption procedure^32,40–43^. The preparation of biochar is based on the pyrolysis of biomass materials, which can be derived from a wide range of algal biomass, poultry litter, forestry waste, activated sewage sludge, and agricultural crop waste raw materials using the thermal process. Biochar has been known to have an efficient uptake capacity, highly abundant, and low-cost sorbent; it has already been used to eradicate heavy metals^44,45^ and organic contaminants^26,31,40,46^ from wastewater. However, many research studies have shown that biochar uptake capacity has been considerably enhanced after modifying chemically or physically mainly due to the increase of the specific surface area, surface functional groups, and pore volume^47,48^.

Biomass can be converted into biochar by thermal pyrolysis, which (according to the life cycle assessment) is more desirable than chemical treatment methods as per environmental and commercial point of view^49,50^. The obtained material can be ground to fine powder using ball milling. This mechanical operation has important implications. Ball milling reduces the particle size while enhancing the specific surface area, which increases the potential sorption sites for contaminants^51^. This mechanical treatment produces nano carbon material from activated carbon and biochar^52^, and improves adsorption performance for heavy metals^34,53^, dyes^54^, and volatile organic compounds (VOCs)^48^.

Wood apple (*Limonia acidissima*) fruit is found in Asian countries like India, Malaysia and Sri Lanka. It is estimated that one seeding tree of about 12 years of age producing about 25–30 tonnes/ha of fruit. One fruit is only containing about 35–50% of edible portion and used for food preparation and medicinal purposes. Its outer shell of this fruit is discarded as agro-waste. The disposal of outer shells directly into the soil may pollute the environment. Several studies have shown that wood apple shell (i.e., biomass, activated carbon, and biochar) is a simple and effective sorbent for removing malachite green dye^55^, Cd (II)^56^ and Cr(VI) ions^57^, iron and fluoride^58,59^, methylene blue^60^, and ibuprofen^61^.

Therefore, the objective of this study was to investigate the new ball-milled biochar, which was prepared from an abundantly available agro-waste wood apple fruit shell material for the elimination of phenol, 4-CPh, and 2,4-DCPh from wastewater. As stated earlier, these phenols and its chlorine derivatives have high toxicity even at low concentrations in wastewater and remain into the environment for longer periods. Biochar characterization was performed using CHN, BET, FTIR, SEM–EDS, TGA, and particle size analysis. Batch adsorption experiments were systematically undertaken in terms of relevant methods such as initial concentration agitation time, biosorbent dose, and solution pH. The adsorption capacity of wood apple fruit shell biochar was experimentally and theoretically investigated using equilibrium kinetics and isotherm models. Thus far, to our knowledge, the adsorption of organic pollutants [e.g., phenol, 4-chlorophenol (4-CPh), and 2,4-dichlorophenol (2,4-DCPh)] by wood apple fruit shell biochar has not been reported in the literature.

## Materials and methods

### Chemicals

Phenol (purity 99.5%), 4-CPh, and 2,4-DCPh (purity 98%) were procured from the Somatco trading company, Riyadh (Loba Chemie Pvt Ltd. India) and used without further purification. Phenol, 4-CPh, and 2,4-DCPh have molecular formulas and weights of C_6_H_5_OH, C_6_H_4_ClOH, and C_6_H_4_Cl_2_O and 94.11, 128.56, and 163.00 g/mol, respectively. To prepare stock solutions, 1 g of phenol and chlorophenols was used by dissolving in 1000 mL of double-distilled water. The working concentration ranges from 100 to 400 mg/L required in our experiments were obtained using the stock solution. Solution pH was set with the help of 0.1 M NaOH and 0.1 M HCl solutions.

### Collection of adsorbent

First, ripen wood apple (Limonia acidissima) fruits shells were collected from the native fields of Venkatareddy palem (Village), Nellore (Andhra Pradesh, India) district. The fruits were washed, dried under sunlight, broken up, and inside pulp was removed. Separated shells were thoroughly washed with distilled H_2_O and sundried for 5 days. Then, dehydrated wood apple shells were chopped into small pieces, dried in an oven at 100 °C for 24 h, and used for biochar production.

### Preparation of wood apple fruit shell biochar

A total of 10 g of weighed, chopped wood apple shell in a ceramic crucible boat was inserted into a horizontal tube furnace of stainless steel having length of 720 mm, 50 mm diameter (Carbolite, UK,) at 700 °C for 4 h, and argon gas was flowed at 80 mL/min. The furnace temperature was gradually increased for 70 min at the rate of 10 °C/min; then, the sample was allowed to cool under argon atmosphere. The collected biochar was ground in a ball milling apparatus (Fritsch, Pulverisette 7 Premium line, Germany) with zirconia ceramic and steel balls at 400 rpm for 8 h. The final ball milling biochar product was named WAS-BC (wood apple fruit shell biochar). WAS-BC was stored in an airtight desiccator and used without any further treatment for the removal of phenolic contaminants.

### Batch studies

Batch adsorption studies were performed using WAS-BC for the elimination of phenolic contaminants from water. The phenols removal was examined at different experimental parameters to study the effect of agitation time, initial adsorbate concentration, sorbent dose, and solution pH (2–10). In a typical experiment, 100–400-mg/L (100 mL) phenol solutions, which were prepared in amber glass bottles (125 mL) and contained 0.1 g of WAS-BC as adsorbent, were agitated on an incubated water bath shaker (30 ± 1 °C) at 200 rpm/min for different periods of times until equilibrium was reached. Under further shaking, the suspension was filtered through 25 mm PTFE 0.22 μm hydrophilic syringe filters (Alwsci Technology, China) to obtain the supernatant solution. To measure the amount of phenol, 4-CPh, and 2,4-DCPh absorbance, a clean solution was analyzed at 270, 280, and 284 nm using a UV–Vis spectrophotometer (Shimadzu UV-1900, Japan). The amount of adsorptive equilibrium capacities and removal rate efficiency of phenols were measured by using Eq. (1) and (2):

1 $${q}_{e}=\frac{\left({C}_{0}-{C}_{eq}\right)V}{m}$$

2 $$\% R=\frac{\left({C}_{0}-{C}_{eq}\right)}{{C}_{0}}100$$

where C_eq_ and C_0_ are the equilibrium and initial concentrations of phenols (mg/L), respectively; q_e_ is the adsorption equilibrium capacity of the adsorbent (mg/g); *R* is the removal efficiency (%), V (L) is the phenol volume; and m (g) is WAS-BC of the adsorbent. The temporal uptake capacities (*q*_*t*_ mg/g) of phenol, 4-CPh, and 2,4-DCPh were computed as:

3 $${q}_{t}=\frac{\left({C}_{0}-{C}_{t}\right)V}{m}$$

where C_0_ and C_t_ in mg/L denote the initial concentration of the pollutant solution and the concentration after adsorption time t (min).

### Validation of kinetic and equilibrium models

The normalized standard deviation $\Delta$ *q*_*e*_ (%) and the Chi-square (χ^2^) given by Eqs. (1) and (2) were used to predict the best fit of kinetic and equilibrium isotherm models. It can be expressed mathematically as shown in the supplementary material section (Text S1).

## Results and discussion

### Characterization of the WAS-BC adsorbent

CHN (Elemental analysis), FTIR, BET, SEM–EDS, XPS, TGA, and particle size analysis were used to characterize WAS-BC (Table 1). The complete physicochemical characterization of WAS-BC was performed to conclude the adsorption technique involved in the elimination of phenol and CPhs compounds.

**Table 1:** Physico-chemical, surface and elemental analysis properties of WAS-BC adsorbent.

Parameter	Value
Color	Black
Odour	None
Moisture content (%)	4.17
Ash content (%)	3.41
BET surface area (m^2^/g)	268.41
BJH cumulative pore volume (cm^3^/g)^a^	0.042
BJH average pore diameter (nm)	4.09
Carbon (%)	81.18
Hydrogen (%)	2.12
Nitrogen (%)	0.12
Oxygen (%)	16.58

^a^Between 1.70-nm and 300.0-nm diameter.

### BET Surface area and pore diameter properties

**Figure 1:**
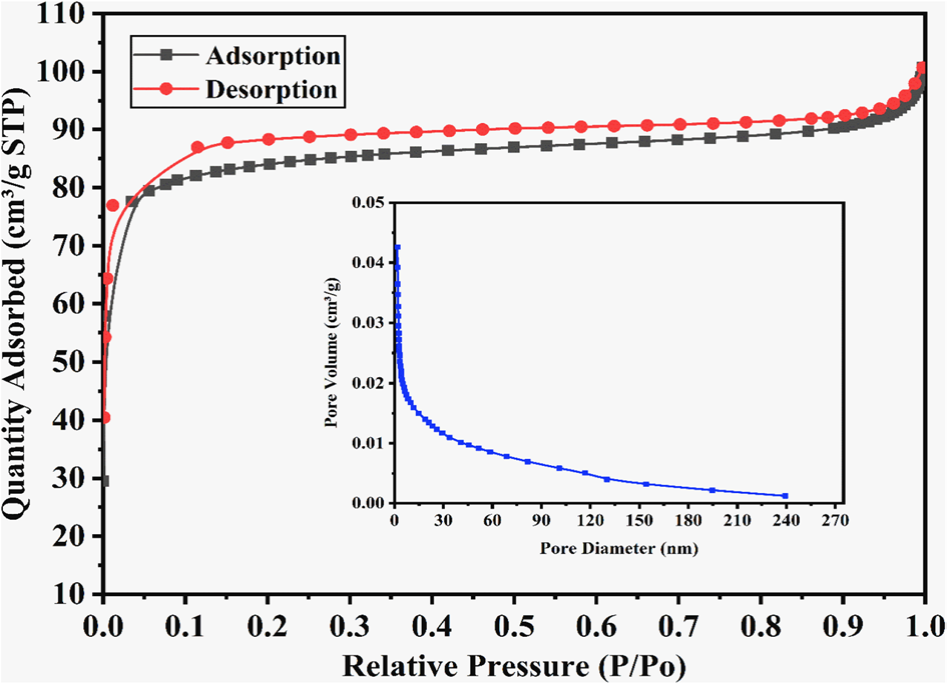
N_2_ adsorption–desorption isotherms and the pore diameter (inset) for WAS-BC.

**Figure 2:**
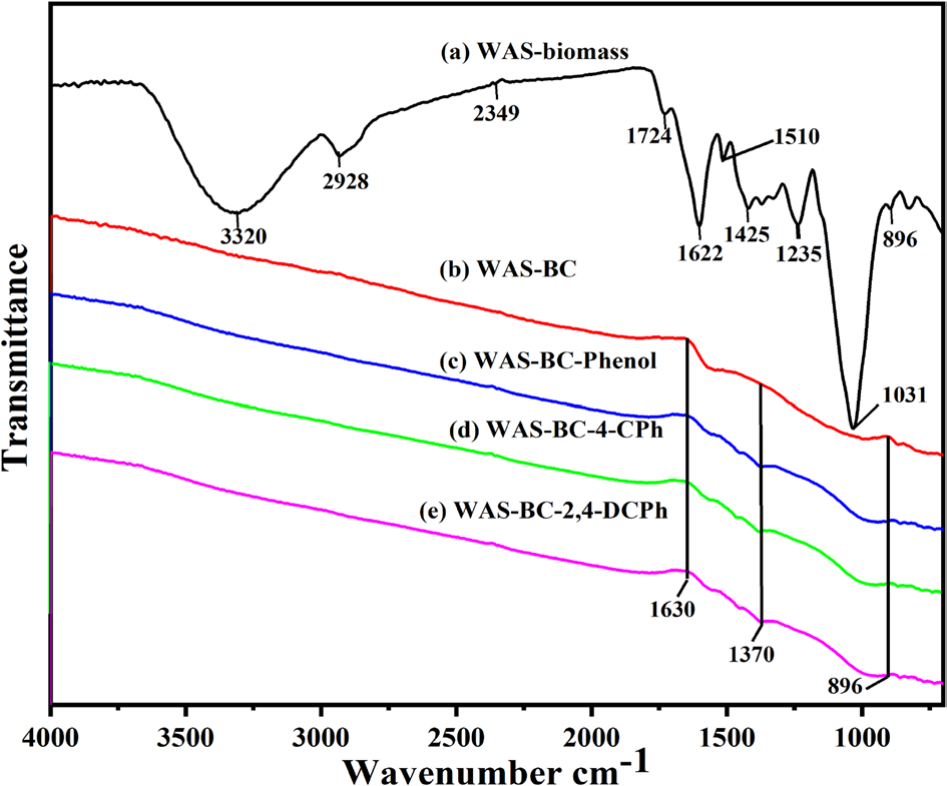
FT-IR spectra of **(a)** WAS-biomass, **(b)** WAS-BC, **(c)** WAS-BC-phenol, **(d)** WAS-BC-4-CPh, and **(e)** WAS-BC-2,4-DCPh.

The WAS-BC adsorbent N_2_ adsorption–desorption isotherms and the pore diameter (inset) are presented in Fig. 1. The WAS-BC sample exhibits the type I adsorption isotherm curve, which suggests the formation of meso and macropores in the adsorbent. The hysteresis loop of H_4_ showing non-limiting adsorption at high relative pressure imply the uniform narrow slit-shaped porosity of WAS-BC^31,62,63^. In addition, the pore diameter and pore volume distributions of WAS-BC (Fig. 2 inset) show that the pores are mostly mesoporous because they fall into the range of 2–50 nm with an average pore diameter size of 4.09 nm. In addition, the total pore volume of WAS-BC was 0.042 (cm^3^/g).

### FTIR analysis

Fourier transform infrared spectroscopy (FTIR) of wood apple shell agricultural biomass waste products and their derived biochar (WAS-BC) was used to explore the potential of using the FTIR technique as a rapid and simple approach for characterizing surface functional groups; the results are displayed in Fig. 2a–e. Figure 2a,b show the spectra of wood apple shell biomass (WAS-biomass) and WAS-BC at 700 °C pyrolysis temperature. As expected, in Fig. 2b, with an increase in the pyrolysis temperature, considerable changes occurred in WAS-BC structural configurations owing to the degree of carbonization^64^. Most major bands disappeared in the WAS-BC sample produced at 700 °C and were replaced by peaks at small wavenumbers, as presented in Fig. 2b. Figure 2a displays that the pristine WAS-biomass sample exhibits a broad peak at 3320 cm^−1^ owing to the existence of O–H bond functional groups corresponding to phenolic hydroxyl groups, while the band at 2928 cm^−1^ is linked to the aliphatic alkyl C–H stretching of hemicellulose and cellulose vibration^61,65^. The O=C=O asymmetric stretching bending vibration is observed at 2349 cm^−1^^58^. The C=O stretching vibration of carbonyl groups is characterized by the peak at 1724 cm^−1^^66^. The higher intensity band at 1622 cm^−1^ is associated to the carbon–carbon double bond of aromatic groups^31^. The bands at 1510 cm^−1^ and 1425 cm^−1^ represent vibrations of the lignin aromatic ring. Amide III bands were observed at 1235 cm^−1^, and C–O–C stretching vibration band was observed at 1031 cm^−1^. In general, the bands below 1000 cm^−1^ are related to hydroxyl and cellulose groups^67^. The peak at 896 cm^−1^ represents the out-of-plane distortion of aromatic C–H atoms^58^. Figure 2c–e show spectra after adsorption; the bands of WAS-BC-Phenol, WAS-BC-4-CPh, and WAS-BC-2,4-DCPh have a considerably smaller intensity than those in the WAS-biomass FTIR spectrum (Fig. 2a). Furthermore, at higher temperatures, many peaks attributed to aliphatic functional groups and carbon–carbon double bond breakages disappeared owing to the availability of sufficient energy. Thus, the prepared (WAS-BC) biochar at higher temperatures had lower (O/C) and (H/C) ratios, which corresponded to the limited existence or absence of surface functional groups and relatively high percentage of carbon^65^.

### SEM–EDS analysis

**Figure 3:**
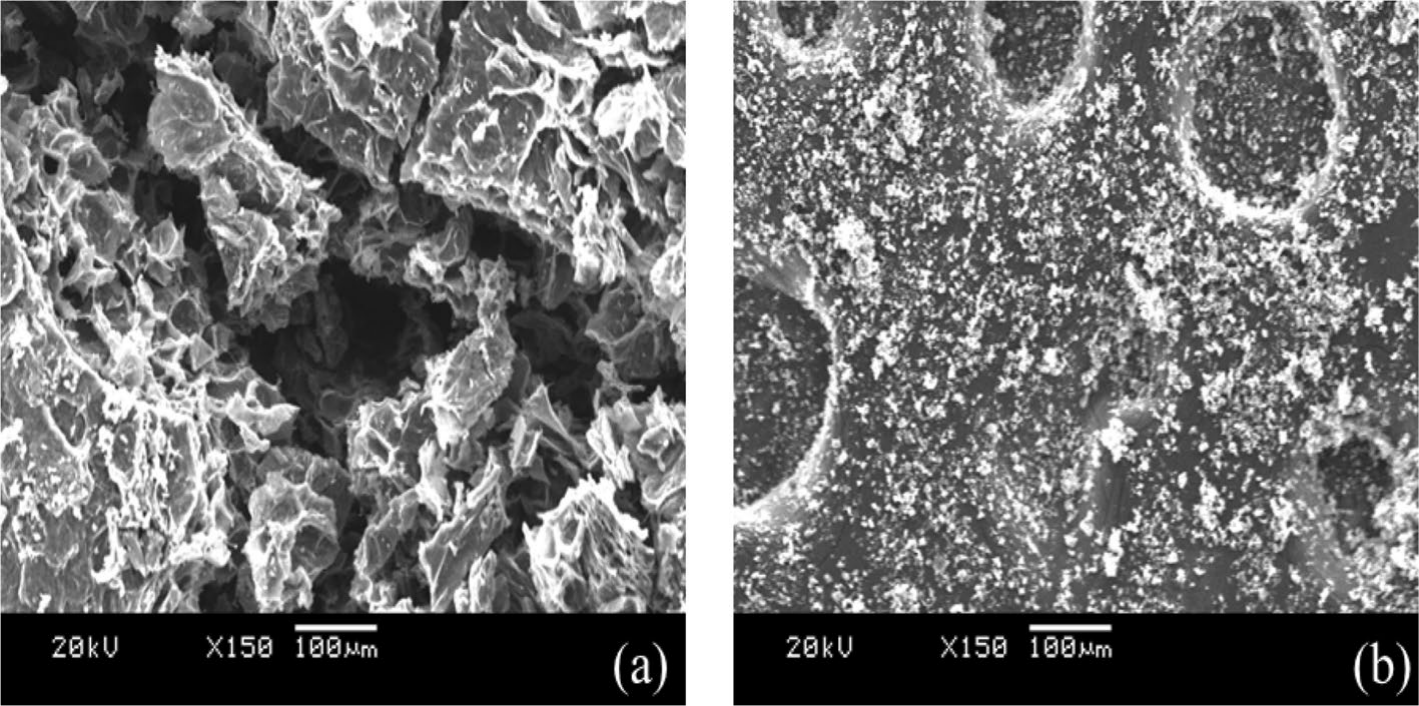
SEM images of **(a)** WAS-BC, **(b)** after ball milling of WAS-BC.

**Figure 4:**
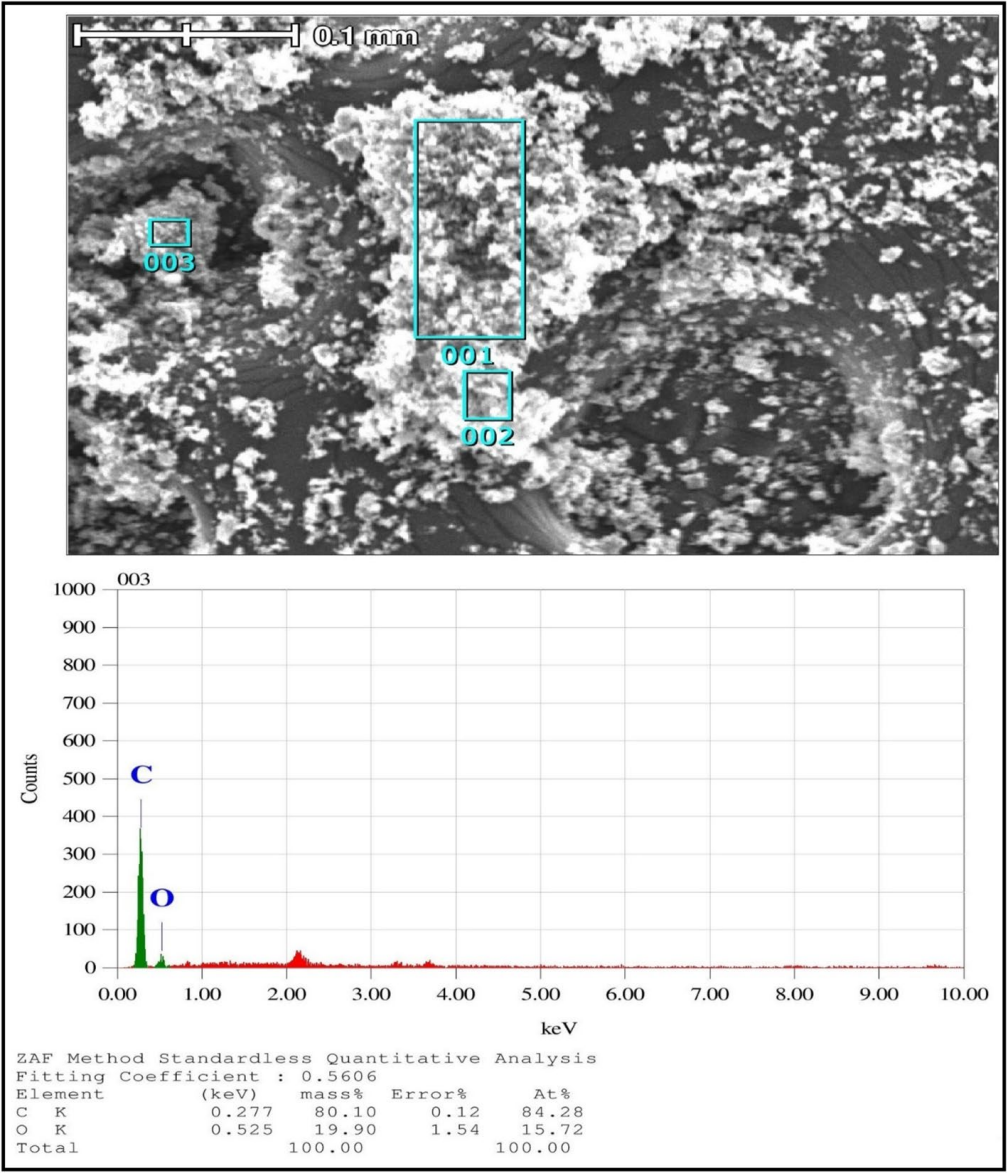
EDS analysis of WAS-BC.

The morphological characteristics of biochar were evaluated by SEM (Scanning Electron Microscopy, JSM-6360A, JEOL, Japan). These samples were sputtered with gold before observation. Figure 3a,b show the appearance of biochar before and after ball milling, respectively. SEM images clearly show that biochar has a porous structure and randomly shaped particles. The appearance of deep porous channels in biochar is owing to the extensive removal of volatile organic material during pyrolysis. It is clear that the reduction of particle size is observed after the ball milling of biochar, which increases the average surface area per unit volume. The average surface area (BET) and pore volume of these samples are 268.41 m^2^/g and 0.042 Cm^3^/g, respectively. The large surface area of absorbent is one of the most important factors in a bio-adsorbent study. The ultimate composition (C, H, N) of biochar is also confirmed by the EDS elemental composition analysis, which is shown in Fig. 4. Although this is a semi-quantitative analysis, the results are determined to be consistent with the C, H, N elemental analysis.

### XPS analysis

**Figure 5:**
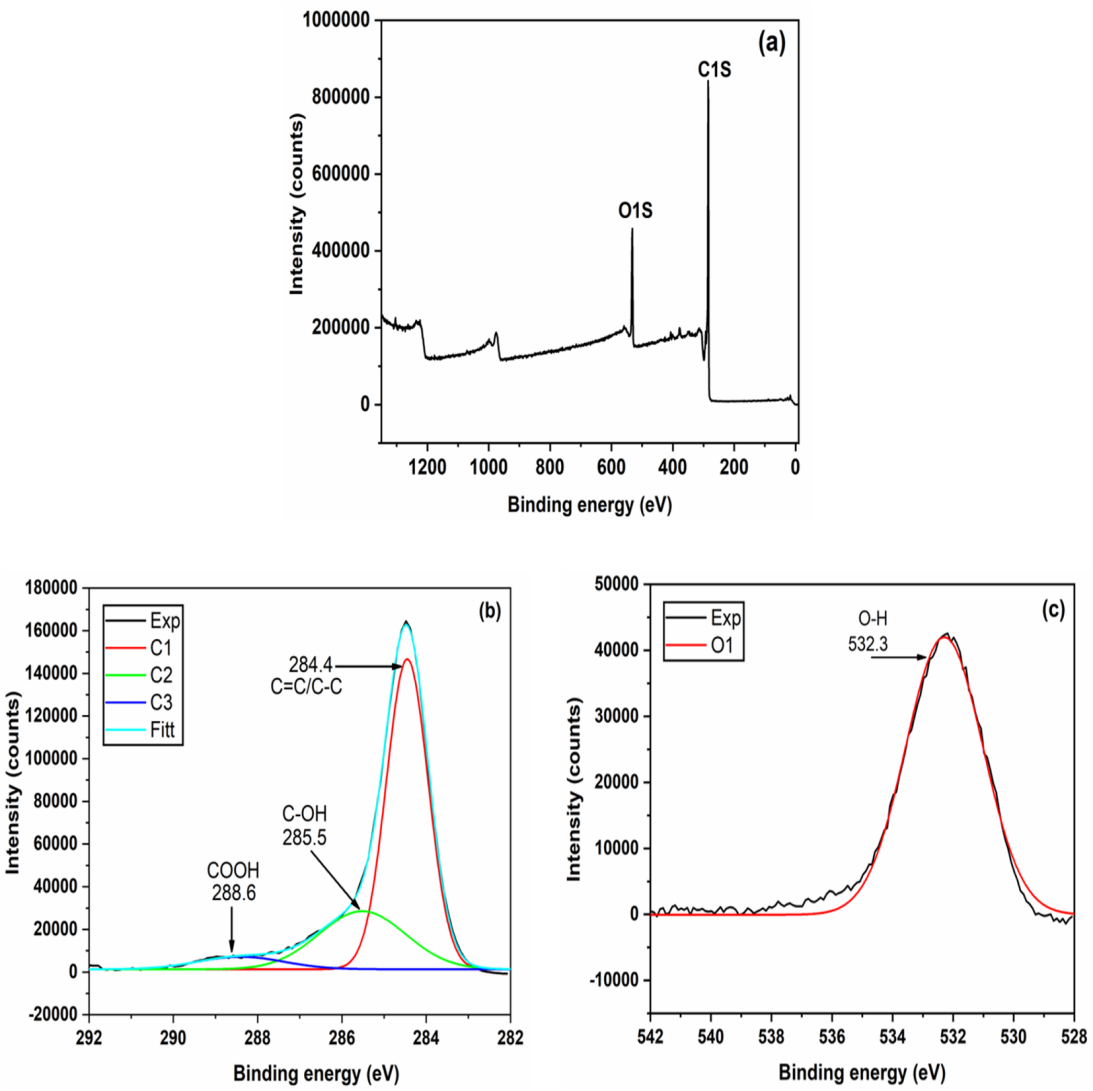
XPS spectra of engineered biochar **(a)** survey scan, **(b)** carbon and **(c)** oxygen element.

The elemental composition and their oxidational states with respective chemical bonds of engineered biochar was determined through the K-alpha X-ray Photoelectron Spectrometer (XPS) (Thermo Scientific, Waltham, MA, USA) analysis and obtained results was depicted in Fig. 5. The Fig. 5a represents the survey scan of engineered biochar and evidenced the presence of carbon and oxygen elements. For better understanding of XPS spectra, the enlarged C1s spectra denotes the 3 peaks at C1 (284.4 eV), C2 (285.5 eV) and C3 (288.6 eV) was observed (Fig. 5b). The peak 284.4 eV represents the C=C/C–C of graphitic or amorphous carbon, peak 285.5 eV attributed to the Sp2 carbon (C=O) and 288.6 eV represents the carboxyl group (–COOH)^68^. Furthermore, the enlarged spectrum of oxygen denotes the peak O1 (Fig. 5c) at 532.3 eV denotes the hydroxyl groups^69,70^. Moreover, the nitrogen was not observed in survey scan (very less quantity/no nitrogen), it is also evidenced by elemental analysis from SEM–EDS analysis.

### TGA analysis

**Figure 6:**
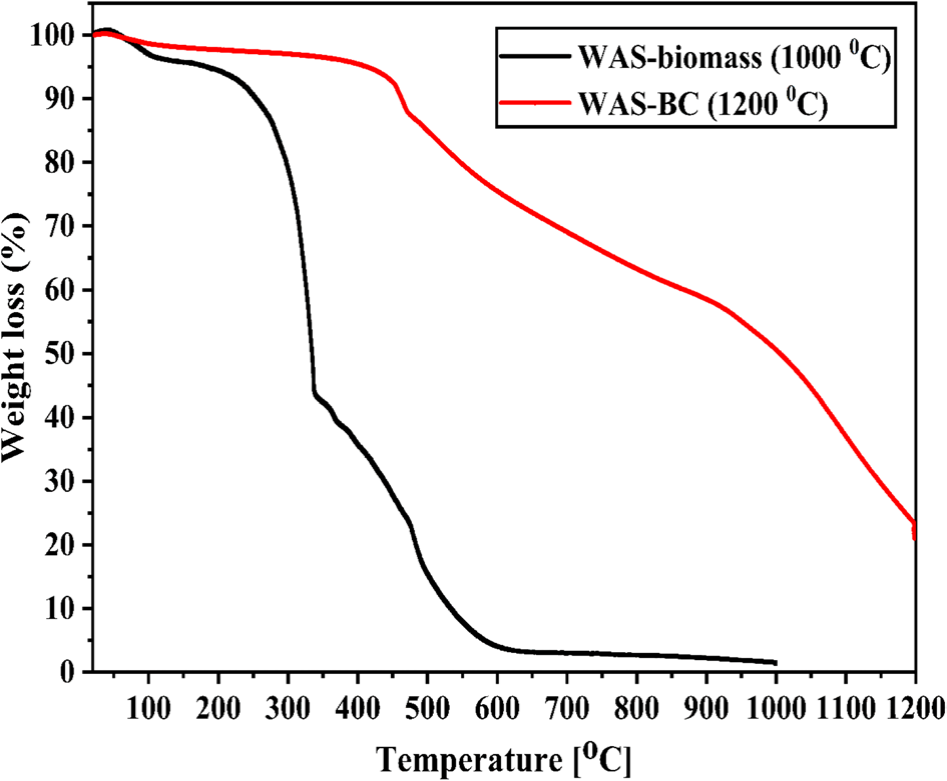
TGA graphs of Wood apple fruit shell biomass and WAS-BC adsorbent.

Figure 6 shows the thermal decomposition of WAS-biomass and WAS-BC evaluated by a thermal gravimetric analyzer (DTG 60H, Shimadzu., Japan). A total of ~ 15–20 mg of neat biomass and biochar was steadily heated at 20 °C/min under an uninterrupted flow of nitrogen. The biomass was heated to 1000 °C, whereas biochar was heated to 1200 °C. It is observed that the evaporation of volatile matter and moisture occurs at 50–100 °C for biomass. Pyrolysis decomposition of this biomass occurs mainly from 200 to 750 °C, and no major changes in mass are observed after this stage. The cracking of aliphatic methylene, methyl, and methoxyl groups with the simultaneous reformation of functional groups (e.g., carbonyl and carboxyl) occur at this temperature range. If biochar is heated to 500 °C, only approximately a 10-wt% mass loss is observed. This can be owing to the evaporation of moisture or adsorbed gases. At higher temperature, the continuous decomposition of biochar is observed owing to extensive carbonization and the subsequent formation of a more graphitic carbon-type structure. Also, the weight loss over a broad range temperature could be attributed to the degradation and decomposition of organic carbon materials in the biochar^71^. Further discrepancy in the estimated stability derives from experimental conditions are observed. This was probably due to the fact the generated biochars have less thermal stability than temperature which they were produced^72^. Also, it is well known that secondary pyrolysis reactions detected and notice if the temperature exceeding the biochars primary decomposition temperature^73^. However, it must be noted that it is stable at wide range of temperature and specifically at our experimental conditions.

### Particle size analysis

**Figure 7:**
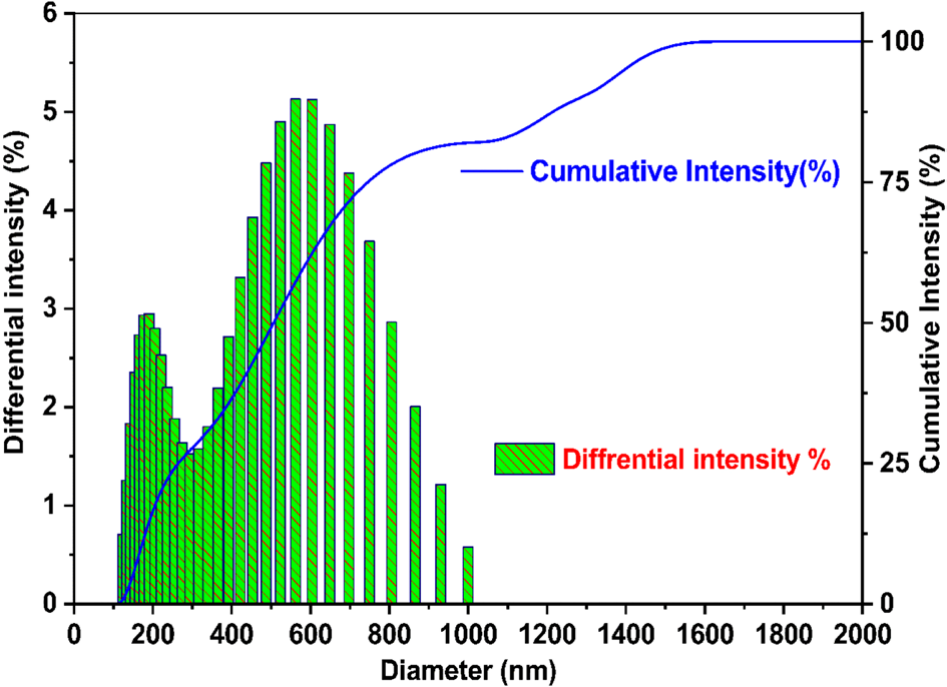
Particle size distribution curve of WAS-BC adsorbent.

Figure 7 shows the particles size distribution of WAS-BC nanoparticles. Most biochar particles are in the range of 110–600 nm, which is considerably smaller than what has been revealed by SEM images. This discrepancy can be explained by the agglomeration phenomenon because strong interparticle forces exist between ultrafine particles with high surface areas. This phenomenon is commonly observed in nanosized particles.

### Effect of solution pH on phenol, 4-CPh, and 2,4-DCPh

**Figure 8:**
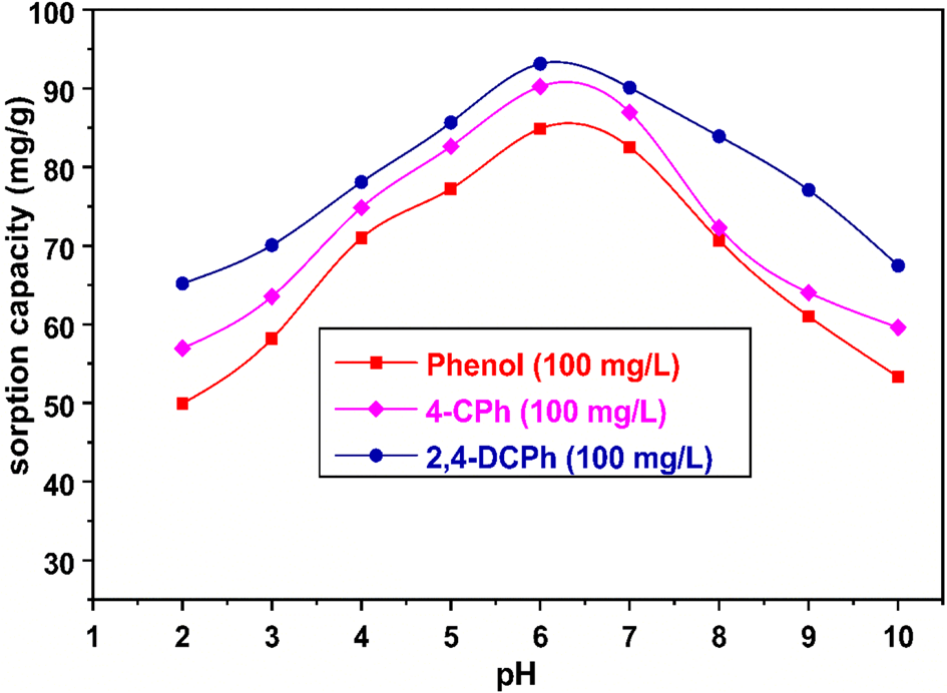
Effect of pH on the adsorption of phenol and CPhs (phenol (filled square), 4*-*CPh (filled diamond), and 2,4-DCPh (filled circle)).

The solution pH value is a significant parameter in the adsorption saturation capacity process. The effect of pH, while other conditions (i.e., initial concentration of adsorbate = 100 mg/L, WAS-BC = 0.1 g/0.1 L, and agitation time = 3 h) remain static, is shown in Fig. 8. As shown in Fig. 8, phenol removal is higher at pH < pKa for each phenol and CPhs compound. Sorption capacity increased with an increase pH of solution from 2.0 to 6.0 and thereafter decreased with a further increase in the pH value. At the optimum pH of 6.0, phenol, 4-CPh, and 2,4-DCPh maximum adsorption capacities were calculated to be 84.87, 90.22, and 93.14 mg/g, respectively. The pKa values of phenol, 4-CPh, and 2,4-DCPh are 9.95, 9.14, and 7.9, respectively. Figure 8 shows that the acidic nature of pH solution in the range of 2–6 is favorable for phenol and CPhs adsorption processes. In aqueous solution, phenols are weakly acidic. For solutions containing more H^+^, the dominant form is molecular state phenols, and the ability to ionize H^+^ is suppressed. At smaller values of pH, protonated phenol and CPhs had higher sorption than their ionizable forms. Moreover, at high pH values, the magnitude of electrostatic repulsion force between the negatively charged adsorbent surface of WAS-BC and phenolate anions/dichlorophenate anions in solution tends to increase^28,32,74^. Clearly, when pH is higher than 6, the adsorption equilibrium decreases. A similar phenomenon has been previously reported for the sorption of phenol and chlorophenols on different agrowaste-based biochar materials such as pine fruit shell biochar (PFS)^32^, pinus massoniana biochar^75^, food waste-based biochar (FWC)^42^, H. fusiformis biochar (HFB)^76^, FA-coated biochars^77^, magnetic biochar^46^, bamboo biochar^78^, and paper sludge/wheat husk biochar^79^. Thus, the optimum solution pH of 6 was chosen for other adsorption experimental studies.

### Effect of agitation time and initial contaminant concentration

**Figure 9:**
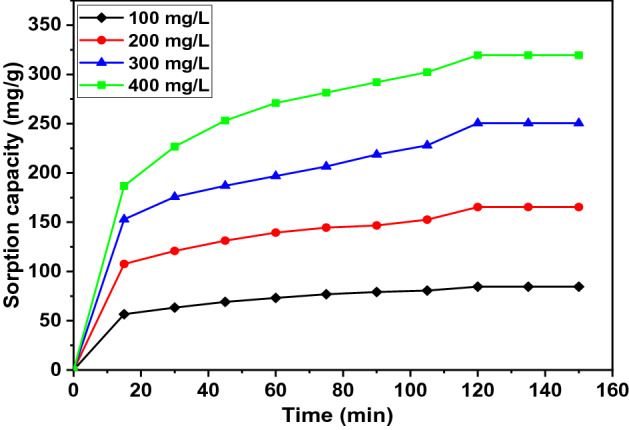
Effect of contact time on the phenol sorption with various initial concentrations, C_0_ = 100 mg/L (filled diamond), 200 mg/L (filled circle), 300 mg/L, (filled triangle) 400 mg/L (filled square).

**Figure 10:**
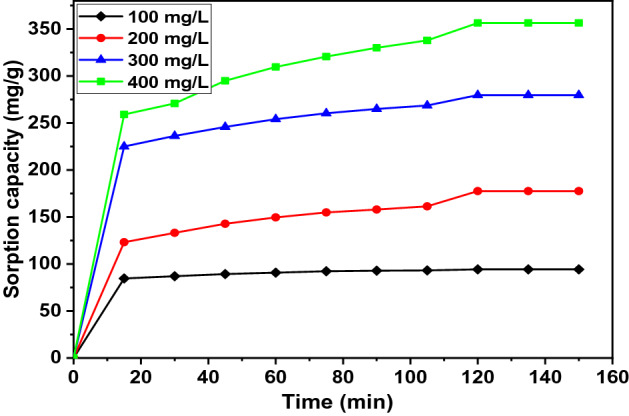
Effect of contact time on 4-CPh sorption with various initial concentrations C_0_ = 100 mg/L (filled diamond), 200 mg/L (filled circle), 300 mg/L, (filled triangle) 400 mg/L (filled square).

**Figure 11:**
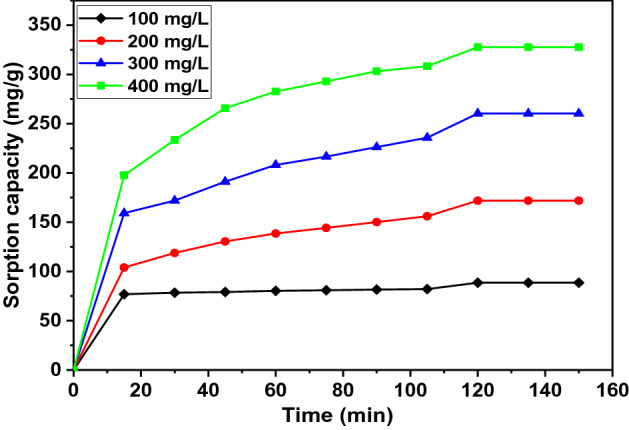
Effect of contact time on 2,4-DCPh sorption with various initial concentrations C_0_ = 100 mg/L (filled diamond), 200 mg/L (filled circle), 300 mg/L, (filled triangle) 400 mg/L (filled square).

Initial adsorbate concentration (of phenol and CPhs compounds) and agitation time are two important parameters that greatly affect the adsorbent uptake capacity. The effect of contact time on the amount of pollutants by adsorption on WAS-BC was investigated at different time intervals up to 15–150 min, 0.1 g/0.1 L of adsorbent, concentrations in the range of 100–400 mg/L of phenol, 4-CPh, and 2,4-DCPh at pH 6.0, temperature 30 ± 1 °C and water bath shaking speed of 200 rpm. The phenol, 4-CPh, and 2,4-DCPh adsorbed capacities on to the adsorbent versus contact time are shown in Figs. 9, 10, and 11. These figures show that sorption capacity increased with an increase in the initial adsorbate phenol, 4-CPh, and 2,4-DCPh concentration ranging from 100 to 400 mg/L. This occurs because the mass transfer driving force between the pollutant and adsorbent increases with an increase in the initial concentration. Figures 9, 10, and 11 shows that the uptake capacity is fast during the initial stages of contact at 15–60 min owing to the large number of available vacant adsorption sites and active sites on the WAS-BC sorbent surface. Then, the adsorption rate of removal slowly increases and reaches an equilibrium state at 120 min. The experiments continued up to 150 min; however, there was no significant enhancement in the uptake capacity. Thus, the optimal agitation time was 120 min.

For comparison, the 120-min agitation time is shorter than those reported for biochar in earlier studies. The phenol removal using food waste-based biochar (FWC 700) reached equilibrium contact time after 24 h for the initial concentration of *C*_0_ = 10–50 mg/L^42^. Shin (2017) has determined that the equilibrium time is 360 min when biochar prepared from *H. fusiformis* (HFB) is used for phenol and heavy metals at the initial concentration of *C*_0_ = 50 mg/L^76^. The removal of phenolic compounds (phenol, 4-CP) using magnetic biochar (MCIB) reaches equilibrium contact time at 420 min for *C*_0_ = 100 mg/L^46^. The removal of 2,4-DCP using paper sludge/wheat husks biochar reaches equilibrium at 120 min and process optimization at 143 min, respectively, for the initial concentration of *C*_0_ = 40 mg/L and 40.28 mg/L^79^. In another study, a 24-h contact time is required for the sorption of pharmaceuticals and halogenated phenols using biochar derived from different agro-waste such as rice straw, fallen leaves, corn stalk, used coffee grounds, and biosolids at the initial adsorbate concentration of *C*_0_ = 50–500 mg/L^74^.

### Effect of adsorbent dose on phenol, 4-CPh, and 2,4-DCPh

**Figure 12:**
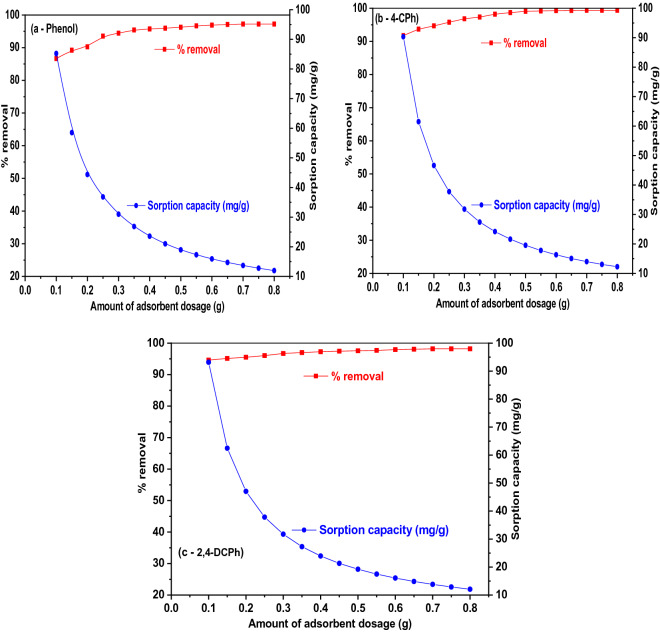
Effect of WAS-BC dose on the sorption of phenol and chlorophenol compounds **(a)** phenol, **(b)** 4*-*CPh, and **(c)** 2,4-DCPh. (WAS-BC dosage = 0.1–0.8 g/0.1 L; phenol and CPh concentrations = 100 mg/L; pH = 6.0; agitation time = 150 min and temp = 30 ± 1 °C).

Adsorbent dose is an important parameter that effects both sorption capacity and percentage removal ratio of phenol, 4-CPh, and 2,4-DCPh by WAS-BC. At fixed solution pH, adsorbate concentration of 100 mg/L, and agitation time of 150 min, the WAS-BC adsorbent dose is 0.1–0. 8 g/0.1 L at 30 ± 1 °C. The results are shown in Fig. 12a–c. Clearly, the obtained results show that the percentage removal ratio of phenol and chlorophenols increase with an increase in the adsorbent dose to 0. 7 g/0.1 L and then levels off; however, the equilibrium amount of uptake capacity gradually decreased. This phenomenon can be ascribed to an increase in surface area and the greater accessibility of active exchangeable sites of adsorbent; then, the equilibrium amount of uptake capacity decreases owing to the saturation of phenol and chlorophenols with a sufficient amount of adsorbent^18,80,81^. Thus, the optimal dose of 0.7 g/0.1 L of WAS-BC is most efficient for the remediation of phenol and chlorophenol compounds.

### Kinetics adsorption studies for phenol, 4-CPh, and 2,4-DCPh

It is important to develop kinetic models to determine the rate, sorption mechanism, and possible rate-controlling step. In this study, the kinetic model adsorption data attained from batch equilibrium studies was investigated using pseudo-first-order (PFO), pseudo-second-order (PSO), intraparticle diffusion (IDM), and Elovich kinetic (EKM) models. PFO and PSO^82^ linear kinetic models can be denoted by Eqs. (4) and (5), respectively

4 $$\mathrm{log}\left({q}_{e}-{q}_{t}\right)=-\frac{{k}_{1}}{2.303}t+\mathrm{log}\left({q}_{e}\right)$$

5 $$\frac{t}{{q}_{t}}=\frac{1}{{k}_{2}{q}_{e}^{2}}+\left(\frac{1}{{q}_{e}}\right)t$$

where q_t_ and q_e_ (mg/g) are the amounts of solute uptake per mass unit of WAS-BC at the equilibrium (mg/g) and time t (min), respectively, and *k*_1_ (1/min) is the adsorption rate intensity constant. The values of *k*_*1*_ and q_e_ can be obtained by plotting log (q_e_ − q_t_) vs. time (t). By plotting t/q_t_ vs. t, *k*_*2*_ (g/mg/min) and q_e_ values can be obtained. For phenol, 4-CPh, and 2,4-DCPh, the resulting kinetic model constant parameters of PFO and PSO as well as other relevant experimental data are shown in Table 2. The obtained results show that PFO experimental q_e(exp)_ disagrees with the q_e(calc)_ values. Accordingly, the PFO kinetic model does not produce the exact sorption results for phenol, 4-CPh, and 2,4-DCPh for WAS-BC. Table 2 shows that the PSO model (R^2^) correlation coefficient values are higher than 0.995, and q_e(calc)_ values are similar to *q*_e(exp)_ values. Therefore, the adsorption kinetic mechanism for WAS-BC can be delineate by the PSO kinetic model.

**Table 2:** PFO and PSO kinetic parameters of phenol, 4-CPh and 2,4-DCPh on WAS-BC.

PFO kinetic model							PSO kinetic model				
Phenol											
Conc (mg/L)	*q*_e,exp_ (mg/g)	*q*_e,cal_ (mg/g)	*k*_1_ (min^−1^)	R^2^	Δq_t_ (%)	χ^2^	*q*_e,cal_ (mg/g)	*k*_2_ (g/mg/min)	R^2^	Δq_t_ (%)	χ^2^
100	84.60	40.59	0.022	0.998	68.37	583.92	88.09	1.0 × 10^−3^	0.999	4.30	0.71
200	165.35	71.81	0.016	0.990	76.40	1786.97	164.58	6.0 × 10^−4^	0.998	3.81	1.05
300	250.47	126.18	0.015	0.980	70.80	1893.00	248.33	3.2 × 10^−4^	0.998	5.65	3.44
400	319.54	181.55	0.021	0.994	59.77	1277.13	322.58	2.1 × 10^−4^	0.999	3.01	1.15
**4-CPh**											
100	88.54	12.48	0.006	0.991	102.84	13,997.42	83.17	6.7 × 10^−3^	0.999	1.48	0.11
200	171.83	85.40	0.015	0.996	71.27	1322.12	171.04	4.7 × 10^−4^	0.998	4.62	1.53
300	260.38	136.46	0.015	0.991	68.63	1724.73	260.76	2.8 × 10^−4^	0.998	6.60	4.88
400	327.74	172.15	0.021	0.995	64.22	1701.24	344.98	2.2 × 10^−4^	0.999	2.91	1.10
**2,4-DCPh**											
100	94.21	15.21	0.026	0.990	95.02	4518.75	95.31	4.0 × 10^−3^	0.999	1.72	0.16
200	177.57	65.34	0.013	0.995	83.93	3204.65	171.78	7.3 × 10^−4^	0.998	3.87	1.22
300	279.65	73.71	0.017	0.998	89.25	7952.93	279.35	6.9 × 10^−4^	0.999	3.23	1.52
400	356.35	140.90	0.018	0.992	77.93	4462.01	360.88	3.2 × 10^−4^	0.998	5.20	4.76

### Intraparticle diffusion model (IPD)

The adsorption process that affects rate-limiting steps can be determined using the IPD model^83^. It can be written as:

6 $${q}_{t}={k}_{id}{t}^{1/2}+C$$

where *q*_*t*_ (mg/g) is the adsorbate uptake mass on the surface of WAS-BC at time *t* (min), *k*_*id*_ is the IPD rate constant (mg/g min^1/2^), and C is the intercept attained from the plot of *q*_*t*_ vs. *t*^*1/2*^. The obtained results are presented in Table 2. The plots do not show a linear trend in the studied time range and do not pass through the origin. Therefore, higher initial contaminant concentrations lead to higher intercept values owing to the mass transport resistance to the process of adsorption, and the boundary layer thickness gradually increased. Therefore, it is determined that adsorption on surface and IPD are involved in controlling the multiple sorption process by WAS-BC of phenol, 4-CPh and 2,4-DCPh.

### Elovich kinetic model (EKM)

The Elovich model has been extensively used to relate the sorption kinetics of contaminants, which describes chemical sorption mechanisms in nature^84^. Moreover, it helps to predict the surface diffusion, mass and activation and deactivation energy of a system. It can be expressed in linearized form as:

7 $${q}_{t}=\frac{1}{b}ln\left(ab\right)+\left(\frac{1}{b}\right)lnt$$

where q_t_ (mg/g) is the uptake capacity at time t; the 1/b (mg/g) parameter is related to the number of active sites accessible for adsorption; a (mg/g/min) is the initial rate of sorption, and b (g/mmol) is related to the adsorption energy. The slope (1/b) and intercept (1/b)ln(ab) constants are obtained from the straight-line plot of q_t_ vs. (ln t). The obtained experimentally calculated *q*_*e*_ values do not fit well with the Elovich model compared with the PSO kinetic model, and the results are shown in Table 3.

**Table 3:** IDM and EKM kinetic parameters of phenol, 4-CPh and 2,4-DCPh on WAS-BC.

Intraparticle diffusion model (IDM)							Elovich model (EM)						
Phenol													
Conc (mg/L)	*q*_e,exp_ (mg/g)	*q*_e,cal_ (mg/g)	*k*_*id*_	C	R^2^	Δq_t_ (%)	χ^2^	q_(*e,cal*)_ (mg/g)	(1/b)ln(ab) (mg/g)	1/b (mg/g)	R^2^	$\Delta$ *q*_*t*_ (%)	χ^2^
100	84.60	82.04	3.86	42.45	0.989	1.22	0.07	80.57	21.04	12.79	0.992	1.20	0.05
200	165.35	154.19	6.98	82.61	0.984	1.50	0.18	151.60	43.57	23.21	0.994	0.92	0.07
300	250.47	226.83	11.36	110.35	0.994	1.01	0.11	222.22	48.90	37.24	0.976	1.96	0.46
400	319.54	308.56	17.73	126.85	0.976	2.68	0.98	302.26	26.24	59.30	0.999	0.44	0.03
**4-CPh**													
100	88.54	82.22	0.81	73.85	0.995	0.14	0.00	81.90	69.40	2.68	0.983	0.30	0.00
200	171.83	157.07	8.06	74.47	0.994	1.06	0.08	153.98	30.08	26.63	0.993	1.18	0.10
300	260.38	236.13	12.48	108.20	0.991	1.43	0.22	230.92	41.53	40.69	0.964	3.04	0.97
400	327.74	318.14	17.56	138.19	0.964	2.95	1.33	312.04	37.81	58.92	0.993	1.24	0.24
**2,4-DCPh**													
100	94.21	93.92	1.41	79.44	0.980	0.49	0.01	93.39	71.56	4.68	0.989	0.38	0.01
200	177.57	163.20	6.13	100.35	0.990	0.93	0.08	160.86	66.43	20.28	0.991	0.99	0.08
300	279.65	270.26	7.01	198.38	0.995	0.41	0.03	267.51	159.97	23.10	0.988	0.72	0.08
400	356.35	339.77	13.08	205.65	0.987	1.18	0.23	334.38	135.39	42.75	0.964	2.06	0.69

The comparison of PFO, PSO, IPD, and EKM model predictions with the experimental data for phenol, 4-CPh, and 2,4-DCPh at various initial concentrations (*C*_0_ = 100–400 mg/L) is shown in supplementary Figures S1(a–d), S2(a–d), and S3(a–d). These figures show that the evaluations of the PSO kinetic model correctly describe the experimental data [q_e_ (mg/g)]. PFO kinetics does not explain sufficiently well the sorption of phenol, 4-CPh, and 2,4-DCPh onto WAS-BC. Moreover, the results fitted with PFO, PSO, IPD, and EKM for contaminant sorption on WAS-BC, their corresponding regression coefficient (R^2^), Chi-square (χ^2^) and Δ*q* (%) values are summarized in Tables 2 and 3. The R-squared (R^2^) values for PSO were greater than 0.998 and were closer to unity than those of other models (i.e., PFO, EKM, and IPD models). By the PSO, the (*q*_*e,cal*_) values exhibit a good fit with the (*q*_*t,exp*_) values owing to the low Chi-square (χ^2^) and Δ*q*_*t*_ (%) values, which were in the range of 0.11–4.88 and 1.48–6.60% for the PSO kinetic model. Therefore, the results of this study show that the PSO model produces a superior fit for phenol and CPhs adsorption onto WAS-BC, which have similar trends; in addition, it is demonstrated that the kinetics of phenol, 4-CPh, and 2,4-DCPh sorption onto other sorbents also followed the PSO kinetic model^19–21,78^.

### Adsorption equilibrium isotherm studies for phenol, 4-CPh, and 2,4-DCPh

Equilibrium adsorption isotherms allow to determine the mechanism of sorption, adsorbent surface properties, and the type of interaction between the sorbent and sorbate. Three adsorption models [i.e., Langmuir, Freundlich, and Dubinin–Radushkevich (D–R)] were fitted with the experimental data at the equilibrium of phenol, 4-CPh, and 2,4-DCPh on WAS-BC^17,85,86^. In addition, these parameters are also expressed mathematically, as shown in supplementary material section Eqs. (3–8) (Supplementary information; Text S2). The three isotherm models with different parameter values are shown in Table 4. The maximum monolayer sorption capacities (Q°_(max)_) for WAS-BC follow the order: 2,4-DCPh > 4-CPh > phenol. A comparable performance was also detected in the study of phenols at the solid–liquid interface of graphene oxide adsorbent with a three-dimensional foam-like structure^31^. The adsorption process may be either chemical or physical in nature depending on the free energy values of *E.* For chemical adsorption, the range of *E* is 8–16 kJ/mol, while the value for the physical nature is 1–8 kJ/mol. Based on the parameter *E* values for phenol, 4-CPh and 2,4-DCPh (10.01, 9.46, and 9.51 kJ/mol, respectively), it can be determined that the chemical nature of the adsorption process is essential for the sorption of phenol and chlorophenols onto WAS-BC. The best fitting linear isotherm models to the experimental data of phenol, 4-CPh, and 2,4-DCPh was evaluated depending upon the higher correlation coefficients (R^2^), lower values of $\Delta$ *q*_*e*_ (%) and χ^2^. To compare the results of three isotherm models (i.e., Langmuir, Freundlich, and D–R), the parameters are shown in Table 4, and the predicted and experimental data are presented in supplementary figures S4, S5, and S6. The results of higher correlation coefficients (*R*^*2*^), lower $\Delta$ *q*_*e*_ (%), and Chi-square (χ^2^) values of Langmuir are compared to those of Freundlich and D–R equilibrium isotherm models. Thus, the obtained experimental data are fit well by the Langmuir isotherm model owing to homogenously distributed active sites on to the WAS-BC surface.

**Table 4:** Isotherm Parameters of 4-CPh and 2,4-DCPh on WAS-BC.

Adsorbates	Langmuir					Freundlich					Dubinin–Radushkevich				
	q_m_ (mg/g)	b (L/mg)	R^2^	$\Delta$ *q*_*e*_ (%)	χ^2^	K_F_ ((mg/g)(L/mg)^1/n^)	n	R^2^	$\Delta$ *q*_*e*_ (%)	χ^2^	q_s_ (mmol/g)	E (kJ/mol)	R^2^	$\Delta$ *q*_*e*_ (%)	χ^2^
Phenol	102.71	0.177	0.998	6.78	3.40	14.834	1.463	0.989	7.69	2.08	4.93	10.01	0.996	4.34	0.81
4-CPh	172.24	0.124	0.999	2.81	0.42	18.360	1.261	0.991	7.71	2.33	4.16	9.46	0.996	4.63	0.93
2,4- DCPh	226.55	0.132	0.998	5.10	1.21	25.321	1.240	0.990	7.57	1.68	3.85	9.51	0.995	5.19	0.90

### Regeneration experiments

**Figure 13:**
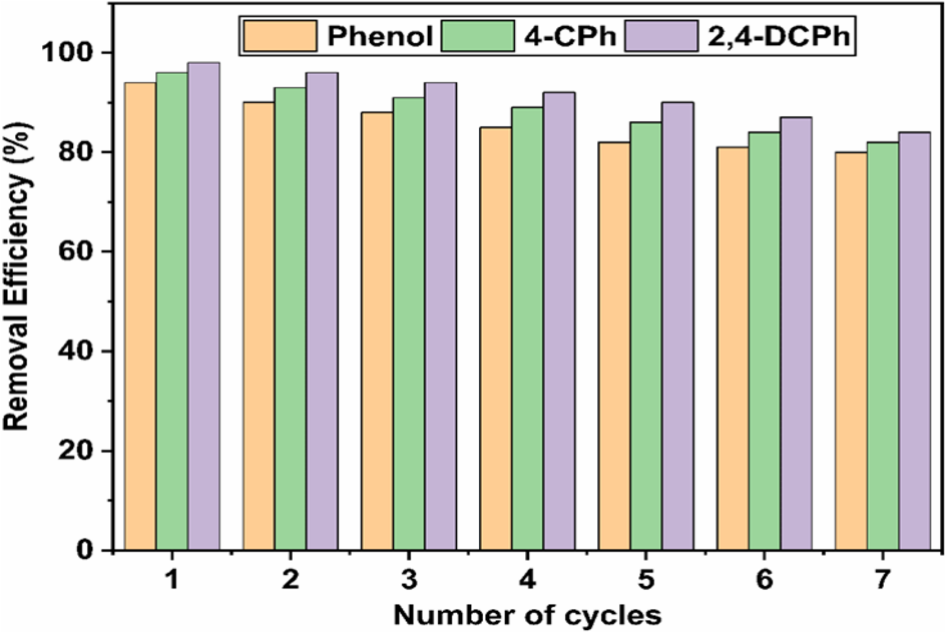
Recycling of WAS-BC biochar for phenol, 4-CPh, and 2,4-DCPh.

The regeneration ability of the biochar is having a vital role for further use an economic point of view. Figure 13 displays the regeneration of WAS-BC over seven cycles of removal efficiency (%) and reusability. The uptake of phenolic pollutants can be recuperated by using 0.1 M NaOH solution^87^. The phenols react with NaOH to form sodium phenolate anion (C6H5O^−^Na^+^) which are readily desorbed phenols from the biochar adsorbent^88^. The removal efficiency of phenol, 4-CPh, and 2,4-DCPh pollutants gradually reduced from the first cycle to seven cycles; though, removal efficiency remained above 82% showing the potential of WAS-BC as a recyclable adsorbent. The used biochar adsorbent can be regenerated and reused upon repeated treatment with 0.1 M NaOH solution.

### Adsorption mechanism

The mechanism of adsorbate-adsorbent interaction in aqueous media is discussed. In general, surface charges are developed upon the dissociation of ions from solution and adsorbent’s surface groups. An adsorbent functional groups and pH of the solution also play important role in adsorption of ions from solution. Moreno-Castilla have proposed three mechanisms such as hydrogen bonding formation, π–π dispersion interaction, and electron donor–acceptor complexation to explain the adsorption of phenolic compounds on to carbon materials^89^.

The large surface area and pore structures of this biochar were observed by BET, and SEM analysis. Similarly, FTIR and XPS analysis suggest that, several functional groups exist on the surface, such as carboxyl, hydroxyl, aromatic rings, and ether groups. Therefore, these functional groups may offer additional sites for phenolic pollutants removal, through hydrogen bonding between phenols and these groups. Also, π–π interaction between the aromatic rings of the biochar and phenols facilitates the removal of phenolic compounds. Moreover, kinetic studies show that, chemical adsorption are dominant factor rather than physical adsorption. The isotherm adsorption study showed monolayer adsorption on a homogeneous sorption surface. The quick initial adsorption characteristic could be ascribed to electrostatic attraction, ion exchange, and chemical adsorption in this study. Such type of adsorption mechanism has been observed in biochar and carbon based adsorbents^88,90^.

### Comparison between WAS-BC with other sorbents

Important literature review for the uptake capacities of phenol, 4-CPh, and 2,4-DCPh is shown in Table 5, which presents sorption capacities achieved by various biochar derived from different biomass adsorbents. It can be inferred that WAS-BC is a capable sorbent for the elimination of phenol and CPhs compounds from contaminated aqueous media.

**Table 5:** The maximum uptake capacities Q^0^ (mg/g), biochar derived from different biomass and the remediation process of phenol and CPhs compounds from aqueous stream.

Biochar	Phenol and CPh compounds, Q^0^ (mg/g)			Conditions					References
	Phenol	4-CPh	2,4-DCPh	pH	Temp	Particle size	Pyrolysis temp (°C)	Residence time	
Chicken manure biochar	106.2	–	–	7	22	2 mm	500	2 h	^9^
PFS (BC550)	26.73	–	–	6.5	25	≤ 1 mm	550	1 h	^32^
FWC700	14.61	–	–	3	35	–	700	–	^42^
MCIB	62.6	131.6	–	7	25	< 75 μm	500	2 h	^46^
Pinus massoniana biochar	46.12	–	–	5	25	60–100 mesh	–	–	^75^
*H. fusiformis* (HFB)	30.09	–	–	6	25	0.5–1.0 mm	550	2 h	^76^
(BC) Bamboo char plus calcium sulfate	–	–	10.69	6	28	38.67 μm	500	4 h	^78^
(HBC) Hydroxyapatite plus Bamboo char plus calcium sulfate	–	–	16.37	6	28	–	500	4 h	^78^
Biopolymer-based biochar	184.21	–	–	5	25	0.200 mm	800	1 h	^91^
Polymer/RS-derived biochar	–	–	25.5–27.8	4.7	25	5 mm	550	4 h	^92^
Fe_5_-GBC_650_ composite	–	250	–	5–9	25	100 and 200 mesh	650	1 h	^93^
BC & BC-MgCl_2_	24.93 & 43.86	–	–	3.6 & 5.5	25	–	630	1 h	^94^
WAS-BC	102.71	172.24	226.55	6	30	110–600 nm (average)	700	4 h	This study

## Conclusions

This study reveals that ball-milled wood apple shell biochar (WAS-BC) is an effective adsorbent for the uptake of organic contaminants (i.e., phenol, 4-CPh, and 2,4-DCPh) from aqueous media. The determined WAS-BC adsorbent BET surface area was 268.41 m^2^/g, and the average particle size was 110–600 nm. The optimum removal was attained at pH 6.0, and the uptake capacity was rapidly attained during the initial stage (15–60 min) and slowly reached equilibrium state within 120 min for phenol and CPhs compounds. Among the four equilibrium kinetic experiment models, PSO described the experimental results the best, which indicated adsorption by chemisorption. The equilibrium uptake capacity showed that the (q_e_ mg/g) experimental and predicted data are better fitted by Langmuir than D–R and Freundlich isotherm models for the elimination of phenol and CPhs. Thus, sorption occurred on uniform sites; thus, the monolayer maximum uptake capacity Q^0^_(max)_ achieved for phenol, 4-CPh, and 2,4-DCPh was 102.71, 172.24, and 226.55 mg/g, respectively. The D–R isotherm adsorption energy (*E*) suggests that the uptake of phenol, 4-CPh, and 2,4-DCPh onto WAS-BC was chemical in nature. Therefore, ball-milled WAS-BC, which is used for the removal of phenol, 4-CPh, and 2,4-DCPh, has a rapid uptake and high sorption capacity; it is environmentally friendly, inexpensive, and readily available; thus, ball-milled WAS-BC is an alternative raw material that can be used to treat contaminant wastewater.

## Supplementary Information

Supplementary Information.
