# Supplementary material for: Engineered biochar from wood apple shell waste for high-efficient removal of toxic phenolic compounds in wastewater
Source: Sci Rep. 2021 Jan 28;11:2586. doi: 10.1038/s41598-021-82277-2 (PMC7844263; doi:10.1038/s41598-021-82277-2)
Supplement: Supplementary file 1 — Supplementary Information. [file 41598_2021_82277_MOESM1_ESM.docx]

**Supplementary materials**

**Engineered biochar from wood apple shell waste for high-efficient removal of toxic phenolic compounds in wastewater**

Nadavala Siva Kumar^1*^, Hamid M. Shaikh^2^, Mohammad Asif^1^, Ebrahim H. Al-Ghurabi^1^

^1^Department of Chemical Engineering, King Saud University, P.O. Box 800, Riyadh 11421, Saudi Arabia

^2^Department of Chemical Engineering, SABIC Polymer Research Centre, King Saud University, P.O. Box 800, Riyadh 11421, Saudi Arabia

^*^Corresponding author: snadavala@ksu.edu.sa, [shivanadavala@gmail.com](mailto:shivanadavala@gmail.com) (Nadavala Siva Kumar)

**Text S1: Validation of kinetic and equilibrium models**

The normalized standard deviation $\Delta$*q_e_* (%) and the Chi-square (χ^2^) given by the eq (1), (2) were used to predict the best fit of the accuracy of the kinetic and equilibrium isotherm models.

| $\Delta q\left( \% \right)=100\times\sqrt{\sum_{i=1}^{N} \frac{\left[ \left( q_{i,exp}-q_{i,cal} \right)/{q_{i,exp}} \right]^{2}}{\left( N-1 \right)}}$ | (1) |
| --- | --- |

where *q_i,exp_,* and *q_i,cal_* are time-dependent investigation data and the corresponding model predictions, respectively, whereas *N* represents the number of data points.

Chi-square analysis can be expressed mathematically as follows,

| $\chi^{2}=\sum_{i=1}^{N} \frac{\left( q_{e, exp}-q_{e,cal} \right)^{2}}{q_{e,cal}}$ | (2) |
| --- | --- |

where *q_e_* and *q_e,model_* (mg/g) is the uptake amount of contaminants achieved experimentally and predicted by the relevant model, respectively.

**Text S2: Adsorption isotherm studies for phenol, 4-CPh and 2,4-DCPh**

The equilibrium adsorption Langmuir isotherm can be written as ^17^:

| $\frac{1}{q_{e}}=\frac{1}{Q^{0}}+\left( \frac{1}{bQ^{0}} \right)C_{e}$ | (3) |
| --- | --- |

where *Q^0^* (mg/g) is the maximum monolayer uptake capacity, q_e_ (mg/g) is the amount of pollutant adsorbed per unit mass of WAS-BC at equilibrium concentration, *C*_e_ (mg/L) is the equilibrium concentration of the solute in the bulk solution, and *b* (L/mg) is a constant associated to the affinity of the binding sites.

Freundlich isotherm model is valid for heterogeneous surfaces and predicts an increase in the concentration of the ionic species adsorbed on the surface of the solid when the concentration of certain species in the liquid phase is increased. It is represented as ^85^:

| $q_{e}=K_{F}C_{e}^{1/n}$ | (4) |
| --- | --- |

The linear form of Eq. (8) is

| ${lnq}_{e}={lnK}_{F}+\left( \frac{1}{n} \right)lnC_{e}$ | (5) |
| --- | --- |

where *K_F_* and *n* are the Freundlich constants related to the uptake capacity and the adsorption intensity of the adsorbent, respectively. Values of *n* > 1 represent favorable adsorption conditions. The isotherm plots of ln*q_e_* versus ln*C_e_* give a linear graph with a intercept *logK_F_* and slope *1*/*n*, from which n and *K_F_*, respectively, can be calculated.

The Dubinin-Radushkevich (D-R) isotherm is commmonly used to describe the adsorption mechanism with a Gaussian energy distribution onto heterogeneous surfaces ^86^. Generally applied to differentiate between chemical and physical adsorption characteristics of solute ions. Its linear form can be expressed in mathematically by Eq. (10);

| ${lnq}_{e}=lnq_{0}-\beta\varepsilon^{2}$ | (6) |
| --- | --- |

The ln *q_e_* versus *ε*^2^ plot gives the slope value of constant *β* (mol^2^/kJ^2^), while the intercept provides the value of the uptake capacity *q_0_* (mg/g). where q_e_ is the amount of phenol and CPhs adsorbed per unit weight of adsorbent (mg/g), the mean free energy sorption E (kJ/mol) indicative of the heat of adsorption, signifying a physical or a chemical adsorption process. *Ɛ* and *E* are can be expressed by Eqs. (11) and (12) respectively;

| $\varepsilon=RTln\left( 1+\frac{1}{C_{e}} \right)$ | (7) |
| --- | --- |
| $E=1/\left( 2\beta\right)^{1/2}$ | (8) |

where *Ɛ* is the Polanyi potential, *C_e_* is the concentration of adsorbate in solution (mol/L) at equilibrium, *T* (K) is the absolute temperature, and *R* is the universal gas constant (8.314 J/mol/K).


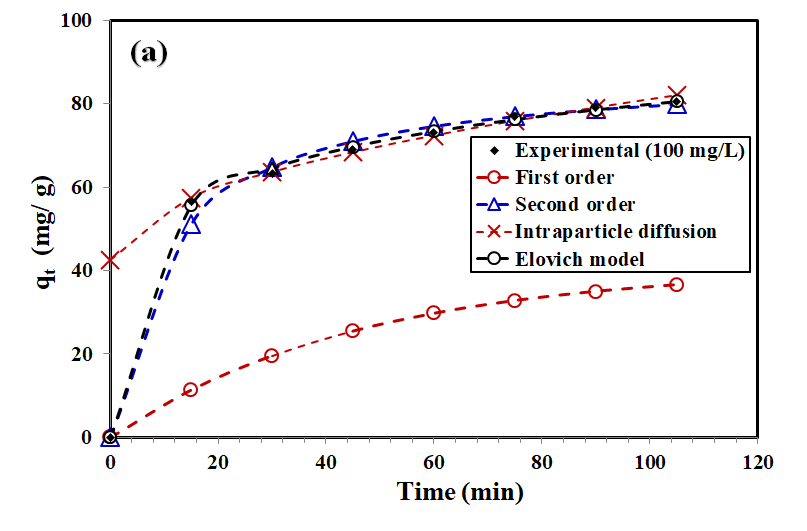


**
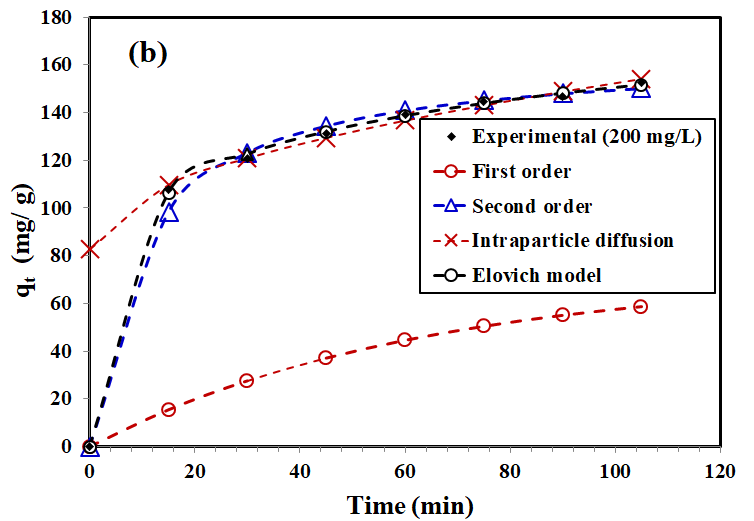
**


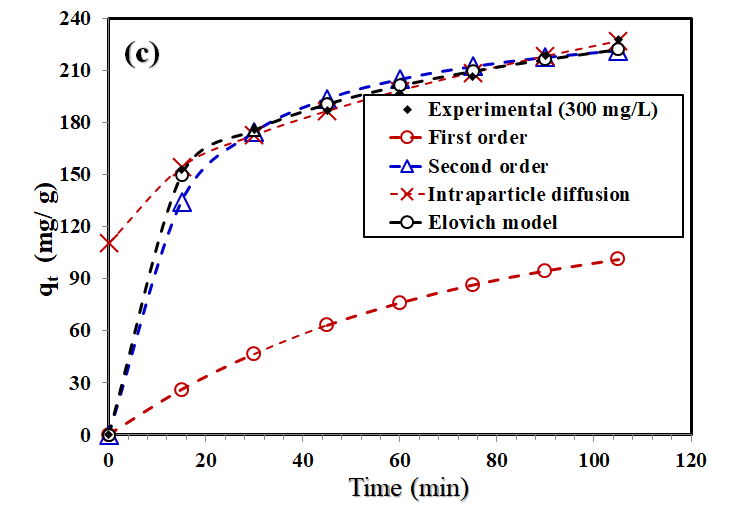


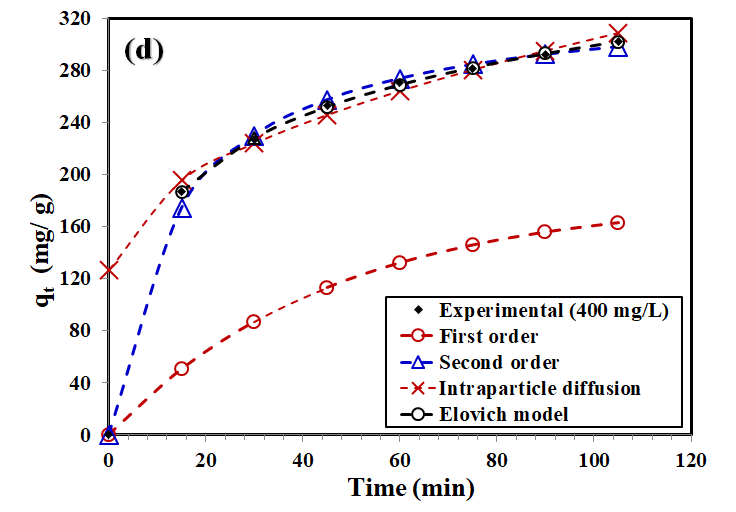


**Figure S1**(**a**–**d**)**.** Phenol Comparison of experimental and calculated values obtained from the PFO, PSO, intraparticle diffusion and Elovich kinetic models [contact time = 150 min; pH = 6.0; agitation rate = 200 rpm; WAS-BC dosage = 0.1g/0.1 L; Temp =30 ± 1°C].


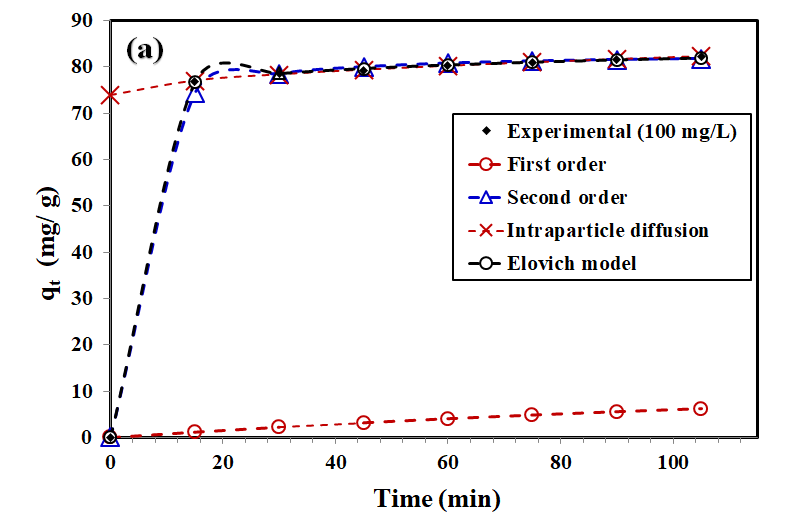


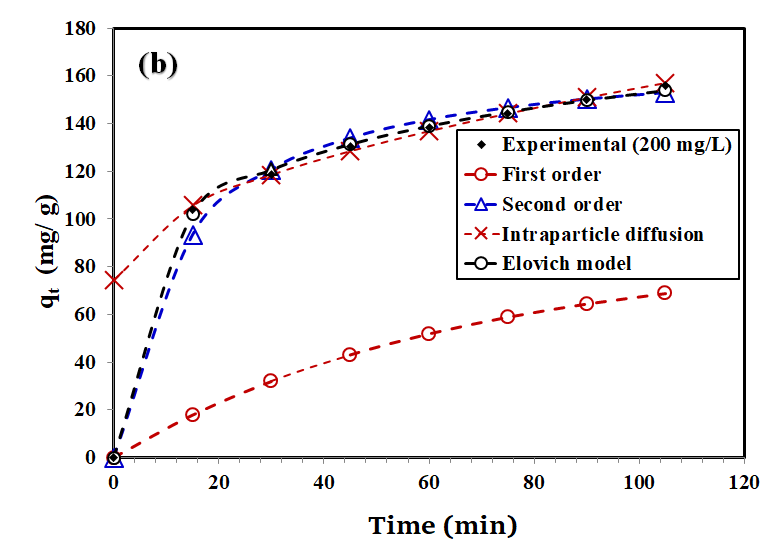


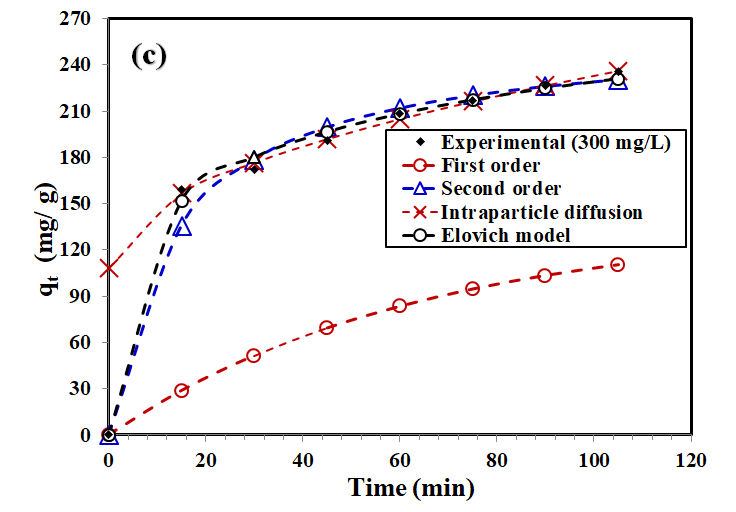


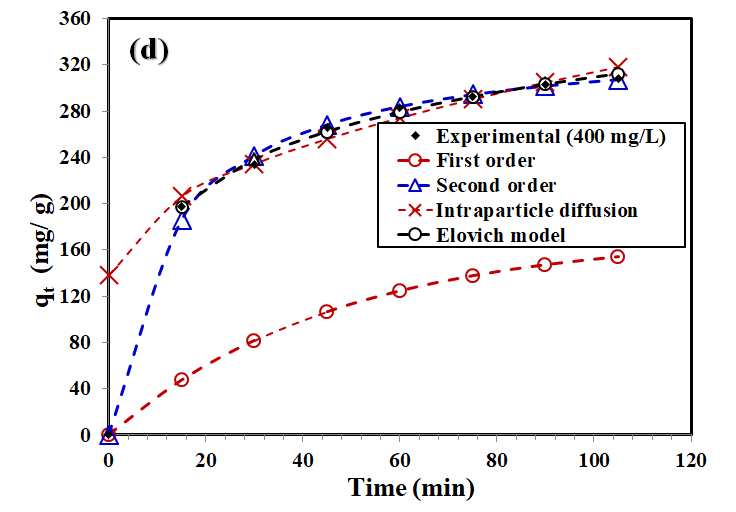


**Figure S2**(**a**–**d**). 4-CPh Comparison of experimental and calculated values obtained from the PFO, PSO, intraparticle diffusion and Elovich kinetic models [contact time = 150 min; pH = 6.0; agitation rate = 200 rpm; WAS-BC dosage = 0.1g/0.1 L; Temp =30 ± 1°C].


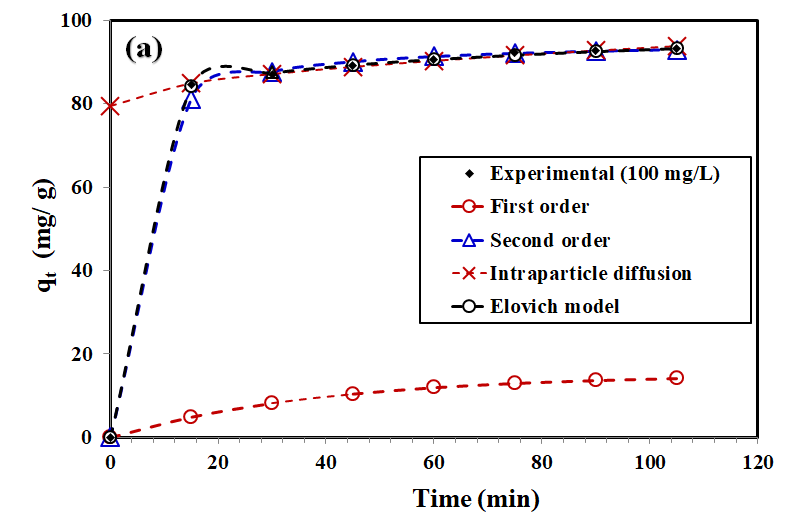


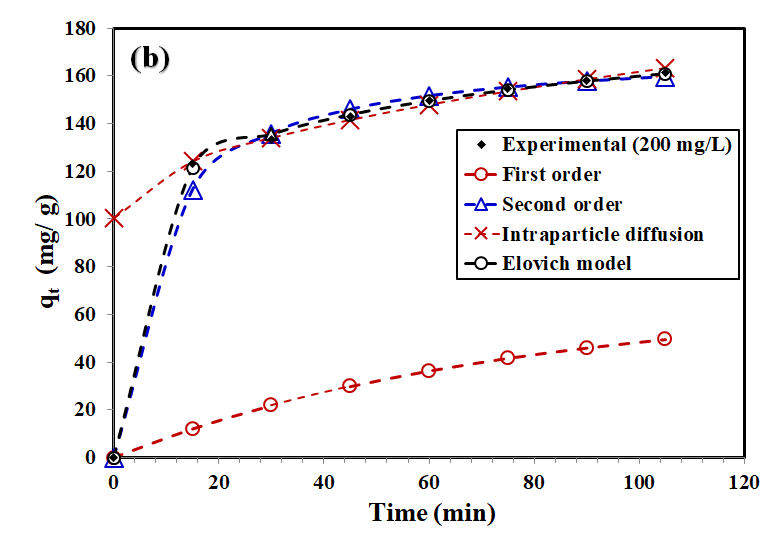


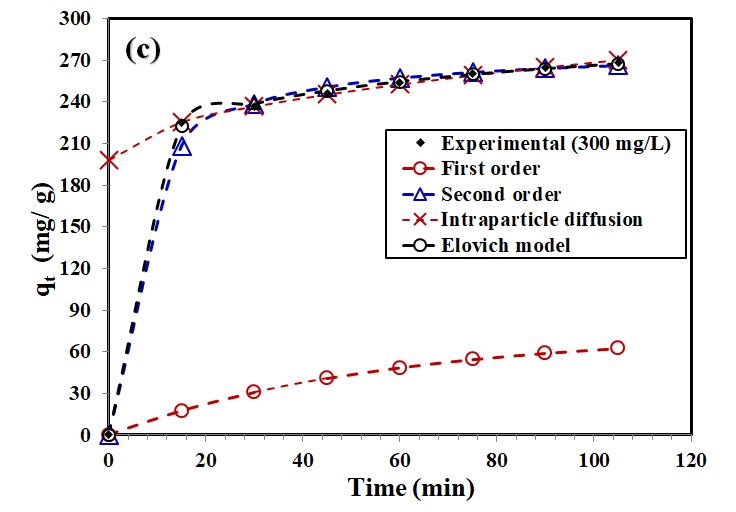


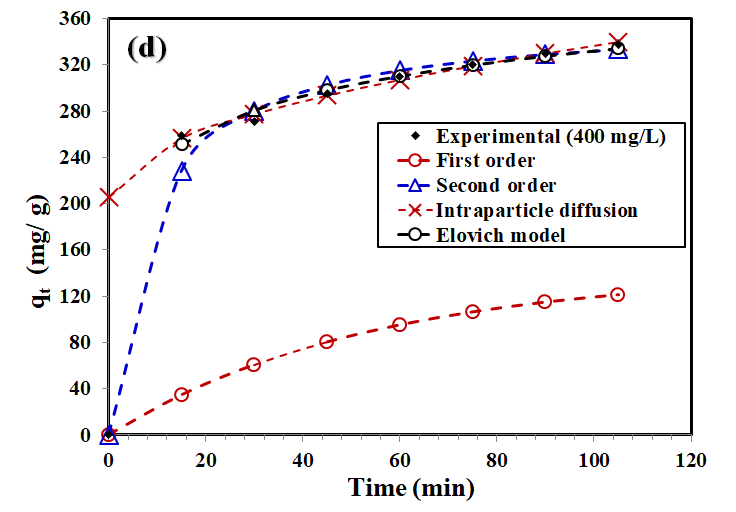


**Figure S3(a–d).** 2,4-DCPh Comparison of experimental and calculated values obtained from the PFO, PSO, intraparticle diffusion and Elovich kinetic models [contact time = 150 min; pH = 6.0; agitation rate = 200 rpm; WAS-BC dosage = 0.1g/0.1 L; Temp =30 ± 1°C].


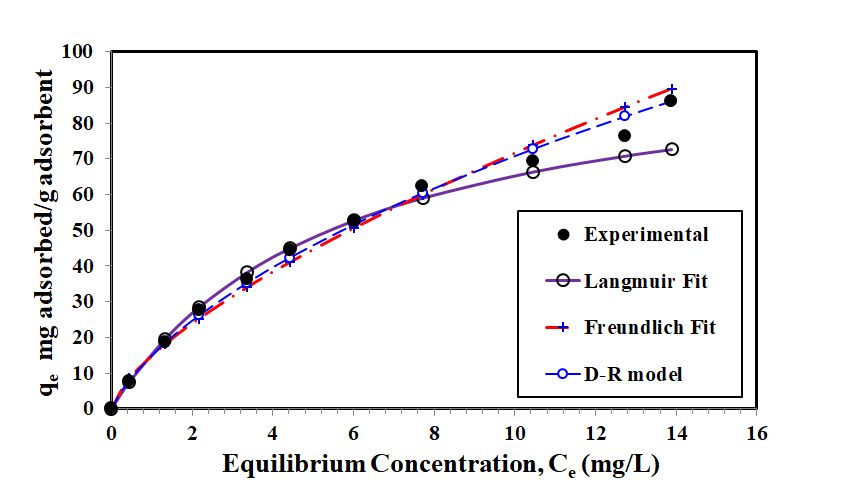


**Figure S4.** Phenol Comparison of experimental and theoretical adsorption parameter values obtained from the Langmuir, Freundlich, D-R and Temkin isotherm models.


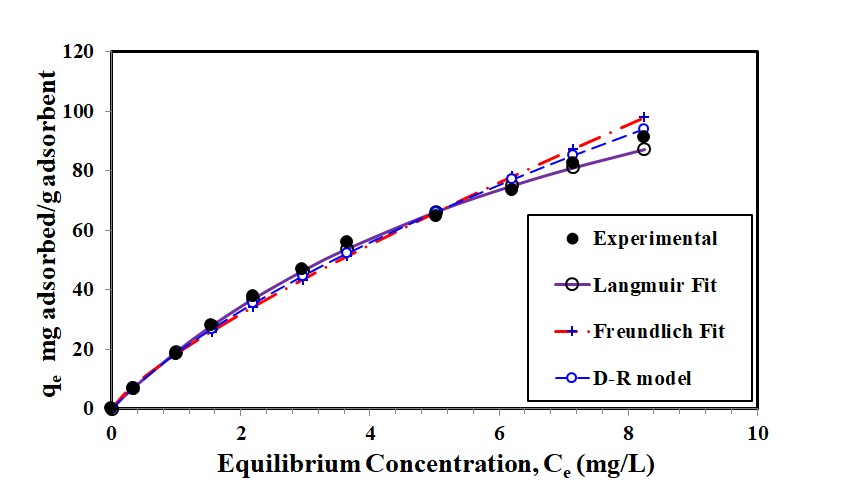


**Figure S5.** 4-CPh Comparison of experimental and theoretical adsorption parameter values obtained from the Langmuir, Freundlich and D-R isotherm models.


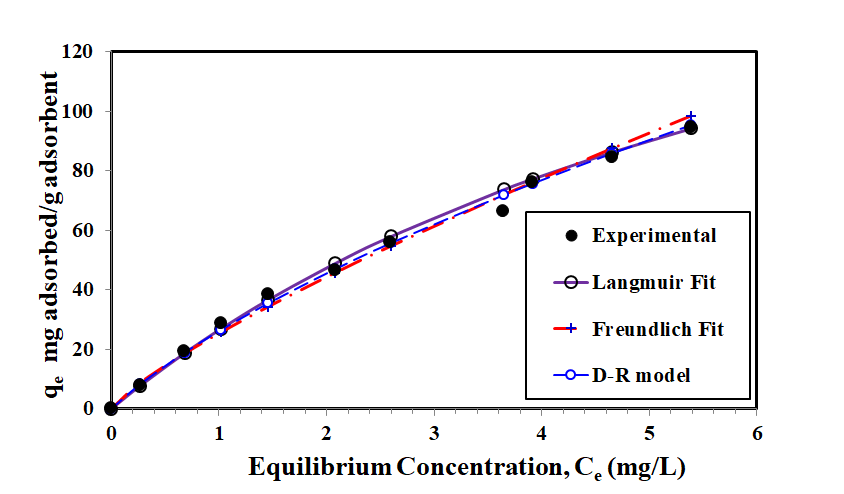


**Figure S6.** 2,4-DCPh Comparison of experimental and theoretical adsorption parameter values obtained from the Langmuir, Freundlich and D-R isotherm models.
